# Découverte d'une tumeur grêlique suite à une occlusion intestinale aigue chez une femme enceinte: à propos d'un cas

**DOI:** 10.11604/pamj.2015.20.340.6324

**Published:** 2015-04-09

**Authors:** Naoufal Chouaib, Mostafa Rafai, Anas El Bouti, Ahmed Belkouch, Hicham El bakkali, Lahcen Belyamani

**Affiliations:** 1 Service des Urgences Médico-Chirurgicales, Hôpital Militaire Mohamed V, Rabat, Maroc

**Keywords:** Grossesse, tumeur côlique, scanner abdominal, pregnancy, celiac tumor, abdominal CT

## Abstract

L'association d'un cancer digestif avec la grossesse est rare et concerne avant tout les cancers colorectaux, de l'estomac ainsi que ceux se révélant par une tumeur de Krukenberg. Le diagnostic est souvent tardif expliquant la fréquence élevée des stades avancés. Nous rapportons un cas d'une femme enceinte qui a présenté une occlusion intestinale aigue sur tumeur colique. Il s'agissait d'une patiente enceinte de 32 semaines d'aménorrhées, admise pour syndrome occlusif évoluant depuis trois jours. L'examen abdominal retrouvait une distension abdominale. L’échographie abdominopelvienne retrouvait une grossesse monofoetale évolutive de 32 SA ± 2SA d'aménorrhée, ainsi qu'un épanchement intrapéritonéal de moyenne abondance. Un scanner abdominal a montré une occlusion grêlique sur processus de la dernière anse iléale. La patiente a été opérée six heures après son admission. Le traitement consistait à réaliser une colostomie temporaire afin de dériver les selles et de décomprimer rapidement le côlon et évacuer les gaz et les selles. Les suites postopératoires ont été marquées par des contractions utérines jugulées par la tocolyse intraveineuse.

## Introduction

Les cancers digestifs découverts pendant la grossesse sont rares [1]. D'une part, la symptomatologie clinique est souvent fruste, car confondue avec les signes dits « sympathiques » de grossesse, ce qui explique le retard diagnostique. Le traitement chirurgical chez la femme enceinte est délicat. Il parait parfois conflictuel quand la guérison de la mère et le développement normal de l'enfant paraissent incompatibles. La sécurité maternelle et fœtale sont prioritaires. Toutefois, le pronostic maternel semble réserver [2]. Nous rapportons un cas d'une femme enceinte chez qui on a découvert une tumeur grêlique suite à une occlusion intestinale aigue.

## Patient et observation

**Figure 1:**
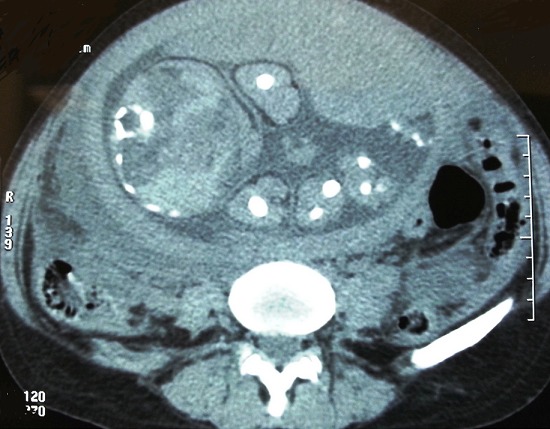
TDM abdomino pelvienne montrant la grossesse associée à l'occlusion digestive

Il s'agissait d'une patiente âgée de 32 ans, primigeste nullipare, enceinte de 32 semaines d'aménorrhées (SA). Elle a été admise pour syndrome occlusif évoluant depuis trois jours. Elle n'a pas d'antécédents médico-chirurgicaux ni d'antécédents familiaux de polypose, ou de cancer digestif, et épidémiologiquement lié (ovarien, endométrial et du sein). La patiente n'a pas présenté des signes digestifs particuliers en dehors des vomissements qu'elle avait attachés à la grossesse. L'examen clinique retrouvait une patiente en mauvais état général, tachycarde à 115 battements par minute, fébrile à 38,2°c. L'examen abdominal retrouvait une distension abdominale sans contraction ni défense. Les orifices herniaires étaient libres. Au toucher rectal, l'ampoule rectale était vide. La biologie standard (ionogramme sanguin, hémogramme, bilan rénal, bilan hépatique) montrait une hypokaliémie à 3,1 mmol/l. L’échographie abdominopelvienne retrouvait une grossesse monofoetale évolutive de 32 SA±2SA d'aménorrhée, ainsi qu'un épanchement intrapéritonéal de moyenne abondance mais ne visualise aucune anomalie hépato-biliaire, pancréatique ou rénale. Devant ce tableau d'occlusion intestinal et le stade avancé de la grossesse, l'indication d'un scanner abdominal a été posée et a montré une distension des anses grêliques avec des niveaux hydroaériques en amont d'un épaississement de la dernière anse iléale associée à des ganglions satellites (Figure 1). Il s'y associe également un épanchement péritonéal de moyenne abondance. Avec un aspect normal du foie, de la rate, du pancréas, des reins et des surrénales. La patiente a été placée à la salle de déchoquage, avec monitorage du pouls, de la tension artérielle, de la saturation artérielle pulsée et du scope. On a procédé à la mise en place d'une sonde naso-gastrique pour soulager la patiente, d'une voie veineuse périphérique avec un remplissage vasculaire par du sérum salé 9 pour mille, et d'une voie veineuse fémorale pour recharge potassique. La patiente a été opérée six heures après son admission. Le traitement consistait à réaliser une hémicolectomie droite emportant la tumeur avec anastomose iléocolique. L’étude anatomopathologique de la pièce opératoire a montré une tumeur carcinoïde. Les suites postopératoires ont été marquées par des contractions utérines jugulées par la tocolyse intraveineuse. La grossesse a évolué normalement et La patiente a accouché à terme, par voie basse, d'un nouveau né bien portant.

## Discussion

Une occlusion digestive est rarement associée à une grossesse. Son incidence réelle n'est pas connue et varie considérablement d'une série à l'autre. Le diagnostic précoce est difficile et ne se fait généralement qu'au cours des complications. Les cancers digestifs représentent une cause rare de l'occlusion digestive au cours de la grossesse. Les TE de l'intestin grêle (fréquemment appelées « carcinoïdes »), le plus fréquemment localisées dans l'iléon distal, représentent dans les séries chirurgicales un tiers de tumeurs de l'intestin grêle [3]. Elles siègent dans 80% des cas au niveau de l'iléon. Les petites tumeurs (moins de 1 cm) sont souvent découvertes par hasard lors d'une laparotomie pour une autre cause et sont traités par une courte résection intestinale [4]. Plus volumineuses, elles sont symptomatiques et s'accompagnent souvent d'un envahissement ganglionnaire et mésentérique. Le traitement associe une résection du grêle, du mésentère et des territoires ganglionnaires régionaux. L'atteinte mésentérique est souvent très importante, imposant une large résection du grêle, disproportionnée par rapport a la taille de la tumeur primitive mais indispensable à une résection curative. Le choix thérapeutique sera alors guide par l’âge du patient, la symptomatologie fonctionnelle, l’évolutivité de la maladie et la longueur du grêle qui restera en fin d'intervention. Parfois l'exérèse est rendue impossible par l'importance de la masse ganglionnaire et de la fibrose mésentérique qui progressent vers la racine du mésentère et englobent les vaisseaux mésentériques supérieurs.

## Conclusion

La rareté de l'association cancer digestif et grossesse rend difficile l’établissement d'un schéma thérapeutique adéquat. La difficulté diagnostique et le retard de la prise en charge peuvent entraîner une importante morbimortalité fœtale et maternelle. D'où l'importance d'une parfaite collaboration entre les différents intervenants: urgentistes, réanimateurs, anesthésistes, obstétriciens, chirurgiens et radiologues.
